# Can *Cynodon dactylon* Suppress the Growth and Development of the Invasive Weeds *Tagetes minuta* and *Gutenbergia cordifolia*?

**DOI:** 10.3390/plants8120576

**Published:** 2019-12-06

**Authors:** Issakwisa B. Ngondya, Anna C. Treydte, Patrick A. Ndakidemi, Linus K. Munishi

**Affiliations:** 1 Department of Sustainable Agriculture, Biodiversity Conservation and Ecosystem Management, School of Life Sciences and Bio-Engineering, The Nelson Mandela African Institution of Science and Technology, Arusha PO Box 447, Tanzania; anna.treydte@nm-aist.ac.tz (A.C.T.); patrick.ndakidemi@nm-aist.ac.tz (P.A.N.); linus.munishi@nm-aist.ac.tz (L.K.M.); 2 Agroecology in the Tropics and Subtropics, Hans-Ruthenberg Institute, University of Hohenheim, 70599 Stuttgart, Germany

**Keywords:** association, Mexican marigold, couch grass, invasion, rangeland, common garden, Eastern Africa

## Abstract

Approaches to managing invasive plants is challenging, particularly in protected areas where conventional methods, such as chemical herbicide applications are limited. We studied the effects of varying densities of *Cynodon dactylon* on the growth and development of the invasive weeds *Tagetes minuta* and *Gutenbergia cordifolia* in northern Tanzania. We conducted pot and field plot experiments following a completely randomized block design that was replicated three times. Increasing densities of *C. dactylon* significantly reduced growth, leaf total chlorophyll, biomass and significantly increased leaf anthocyanin of both *T. minuta* and *G. cordifolia* invasives. Our results further showed that the critical density of *C. dactylon* to suppress the two invasive species is ≥ 8 plants/m^2^. We suggest that *C. dactylon* can successfully be used as an alternative eco-friendly and sustainable approach for managing invasive weeds, such as *T. minuta* and *G. cordifolia*. This management technique can additionally improve forage production and biomass for wild and domestic herbivores in the affected areas.

## 1. Introduction

For many decades, weed management in both farmlands and rangelands has mainly been employed through chemical herbicide applications [1]. This chemical control of invasive weeds has become a normal practice, but it often has been associated with a consecutive evolution of resistant weeds, and hence, further proliferated the problem of weed control [2]. Weed resistance to chemical herbicides is becoming a serious and increasingly challenging issue, further being fueled by heavy reliance on chemical herbicides. Likewise, rising concerns on environmental safety and increasing costs associated with chemical herbicides have highlighted the need for alternative tools that can be used to suppress invasive species. These management strategies need to replace chemical herbicides and be able to reduce the increasing weed resistance, environmental pollution and associated costs.

Management of invasive weeds in protected ecosystems poses great challenges as herbicides, which often have proven successful in farmlands are not recommended for protected areas. Herbicides often have strong negative effects on other native flora and fauna [3,4], while traditional methods of controlling weeds, such as mechanical uprooting, cultivation and burning [5,6] are labor-intensive and costly. Prior to the late 1800s only mechanical control of weeds was used in agriculture [1], but recently, weed control mechanisms, such as biological control and Integrated Pest Management (IPM) have been recommended to complement and improve the traditional control methods [7]. Biological control of weeds, i.e., using living organisms, is aimed at improving ecosystems and reducing the competitive ability of target weed species so as to reduce stress on native plant communities and associated herbivores [8].

Although alternative management options, such as uprooting and mowing are often opted for, they are labor-demanding, and only a short term remedy as many invasive plant seeds remain in the soil seed bank. We propose that plant density-dependent competitive interactions can be used to suppress invasives, particularly the problematic weeds *T. minuta* and *G. cordifolia*. This natural suppression can even help in the restoration of previously invaded ecosystems, particularly grazing areas, by providing healthy forage vegetation for mammalian herbivores. As a low-cost, low-impact management technique, plant-plant competition has been reported to be effective in some restoration projects [9].

We aimed at utilizing *C. dactylon* as a competitor, due to its agronomic value as a forage species [10]. Also, this species was found to be highly competitive in previous studies [11], due to its ability to form deep roots [10], and its ability to grow on soils with a wide range of pH [12]. We studied the density-dependent competitive effect of *C. dactylon* on growth parameters and leaf pigments of two species, i.e., *T. minuta* and *G. cordifolia*, which have recently invaded protected lands of Tanzania. We set up screen house and field plot experiments by varying *C. dactylon* densities and hypothesized that this species would suppress the two weeds by reducing their growth and development. Our study might pave the way for the application of *C. dactylon* as a management tool to suppress *T. minuta* and *G. cordifolia* in invaded rangelands and to improve pasture production and environmental sustainability.

## 2. Results

### 2.1. Invasive Plant Growth Parameters

**Figure 1:**
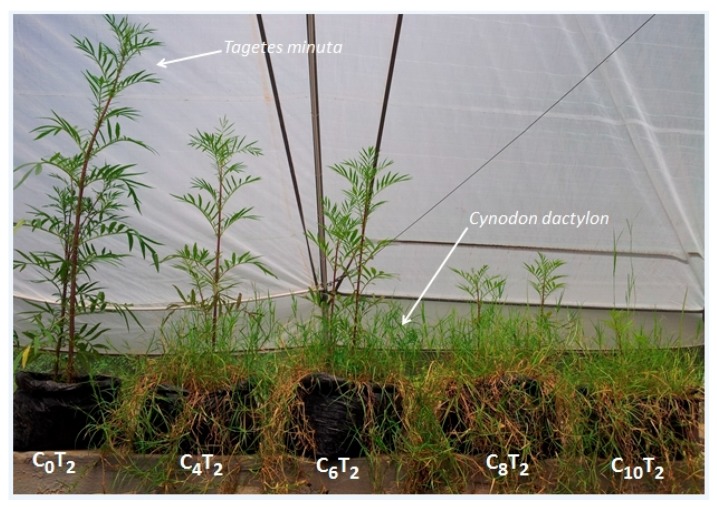
Effects of increasing densities of *Cynodon dactylon* plant individuals on *Tagetes minuta* vigor (C = *C. dactylon* and T = *T. minuta*, numbers represent proportions of sown *C. dactylon* and *T. minuta*, whereby C_0_T_2_ was the control).

**Figure 2:**
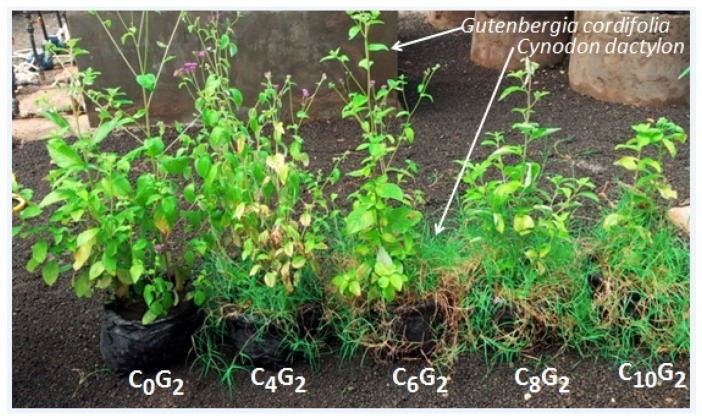
Effects of increasing densities of *C. dactylon* on *G. cordifolia* vigor (C = *C. dactylon* and G = *G. cordifolia*, numbers represent proportions of sown *C. dactylon* and *T. minuta*, where C_0_G_2_ was a control).

We observed a general decrease in *G. cordifolia* and *T. minuta* vigor with an increasing density of *C. dactylon* plants (Figure 1 and Figure 2).

**Figure 3:**
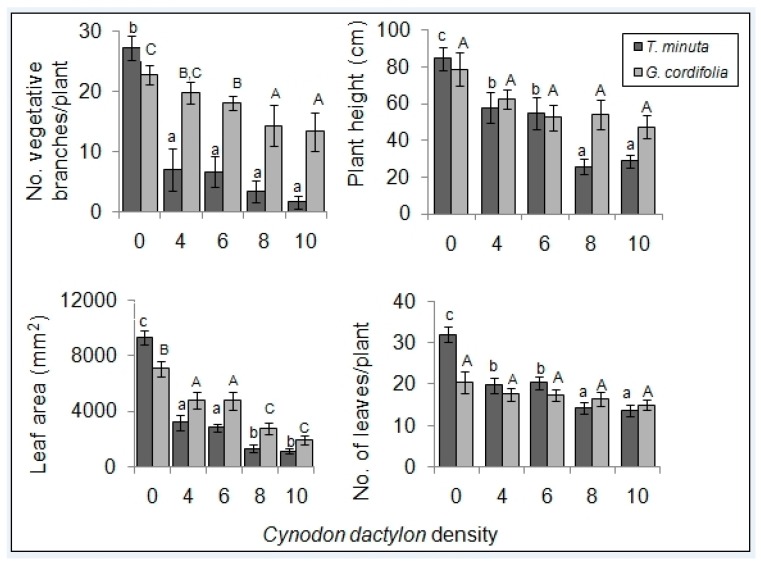
*C. dactylon* varying density effects on the number of vegetative branches, plant height, leaf area, and number of leaves of *T. minuta* and *G. cordifolia* in a screen house (pot) experiment. *C. dactylon* densities ranged from 0 to 10 individuals per pot, while the number of weeds per pot remained constant at two. Bars with dissimilar letters indicate significant differences in the means by Fisher LSD at *p* = 0.05.

**Figure 4:**
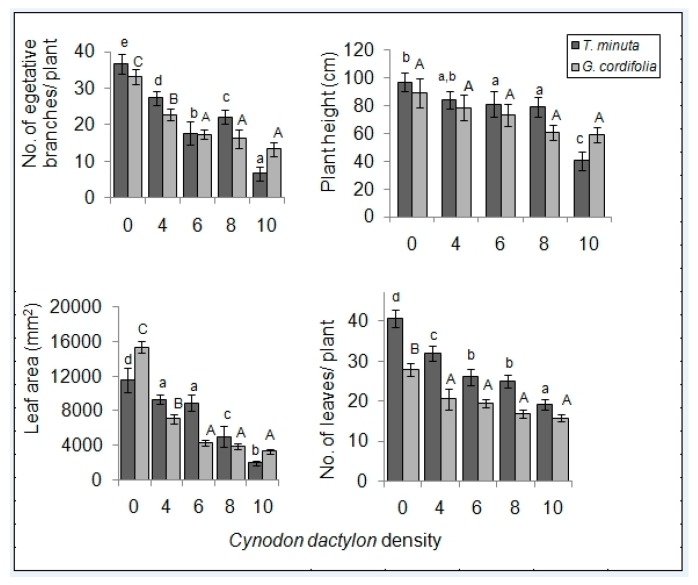
*C. dactylon* varying density effects on the number of vegetative branches, plant height, leaf area, and number of leaves of *T. minuta* and *G. cordifolia* in the field plot experiment. *C. dactylon* densities ranged from 0 to 10, while the number of weeds per plot was two. Bars with dissimilar letters indicate significant differences in the means by Fisher LSD at *p* = 0.05.

The mean number of vegetative branches, panicles and leaf area of both *T. minuta* and *G. cordifolia* species differed significantly across the five *C. dactylon* treatments (Table 1 and Table 2) and was over four times higher in control pots/plots than in pots/plots with *C. dactylon* at densities of >8 individuals per pot/plot (Figure 3 and Figure 4).

The mean number of leaves and plant height differed significantly in *T. minuta* (*p* < 0.05), being half as many and shorter in pots/plots with ≥ 8 *C. dactylon* per pot/plot compared to those of the control. No significant difference was observed for *G. cordifolia* (*p* > 0.05) (Figure 3 and Figure 4).

**Figure 5:**
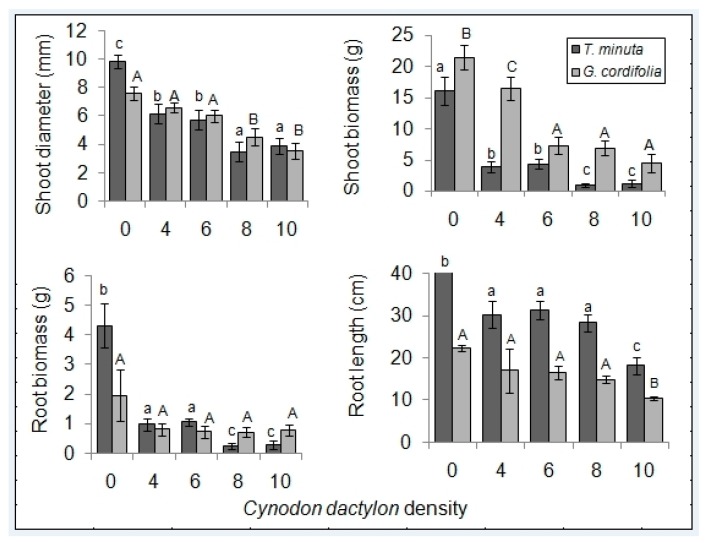
*C. dactylon* varying density effects on mean (±SE) shoot diameter, shoot biomass, root biomass, and root length of *T. minuta* and *G. cordifolia* intercrops in a screen house (pot) experiment. *C. dactylon* densities ranged from 0 to 10, while the number of weeds per plot was two. Bars with dissimilar letters indicate significant differences in the means by Fisher LSD at *p* = 0.05.

**Figure 6:**
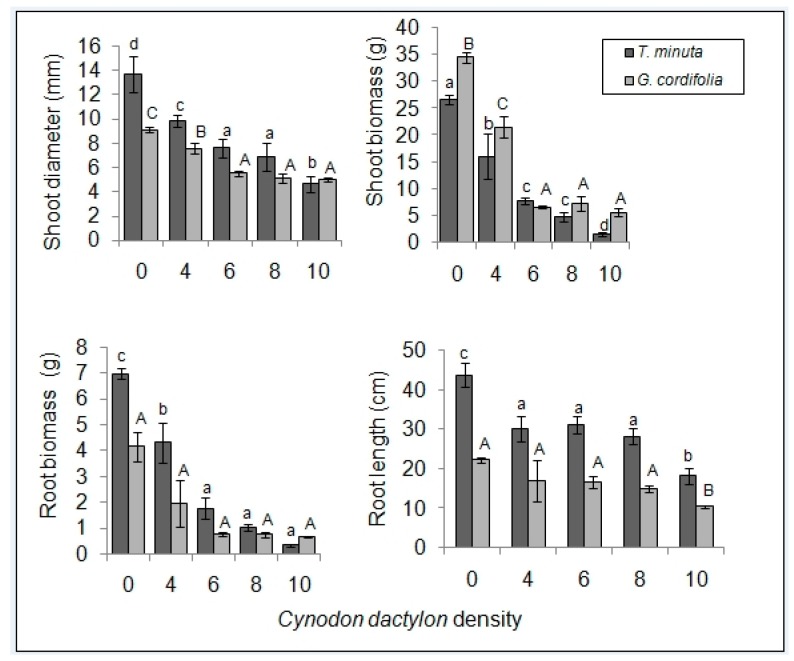
*C. dactylon* varying density effects on mean shoot diameter, shoot biomass, root biomass, and root length of *T. minuta* and *G. cordifolia* intercrops in field plot experiment. *C. dactylon* densities ranged from 0 to 10, while the number of weeds per plot was two. Bars with dissimilar letters indicate significant differences in the means by Fisher LSD at *p* = 0.05.

Mean shoot diameter and shoot biomass differed significantly across the five *C. dactylon* density treatments in both *T. minuta* and *G. cordifolia* (*p* < 0.05). *T. minuta* and *G. cordifolia* in pots/plots with *C. dactylon* density ≥ 8 per pot/plot had half the diameter and were half as heavy as shoots/biomass in control pots/plots. Mean root biomass and root length differed significantly only in *T. minuta* (*p* < 0.05), but not in *G. cordifolia* (*p* > 0.05) (Figure 5 and Figure 6). *T. minuta* root weight in pots/plots with *C. dactylon* density ≥ 8 per pot/plot were over four times lighter in weight and half the length compared to roots of *T. minuta* in control pots/plots (Table 1 and Table 2).

### 2.2. Leaf Pigmentations 

**Figure 7:**
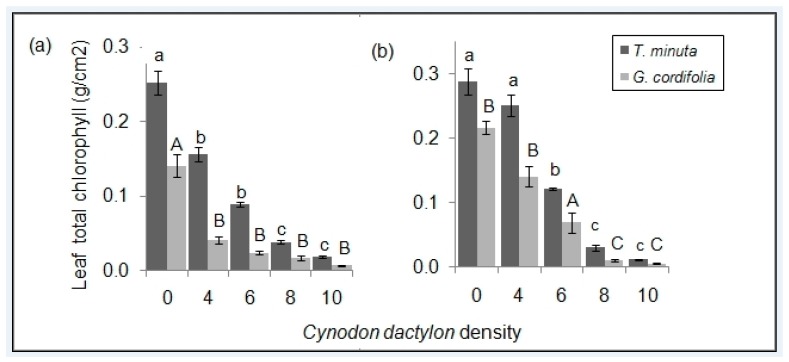
Mean (±SE) total leaf chlorophyll content of *T. minuta* and *G. cordifolia* planted with various *C. dactylon* densities (**a**) in the screen house and (**b**) field plot experiments. *C. dactylon* densities ranged from 0 to 10, while the number of weeds per pot/plot was two.

In both *T. minuta* and *G. cordifolia*, total leaf chlorophyll content differed significantly across the five *C. dactylon* density treatments (*p* < 0.05) (Figure 7; Table 1 and Table 2). *T. minuta* and *G. cordifolia* in control pots/plots had three times higher leaf chlorophyll contents than *T. minuta* and *G. cordifolia* in pots/plots with *C. dactylon* density ≥ 8 per pot or plot.

**Figure 8:**
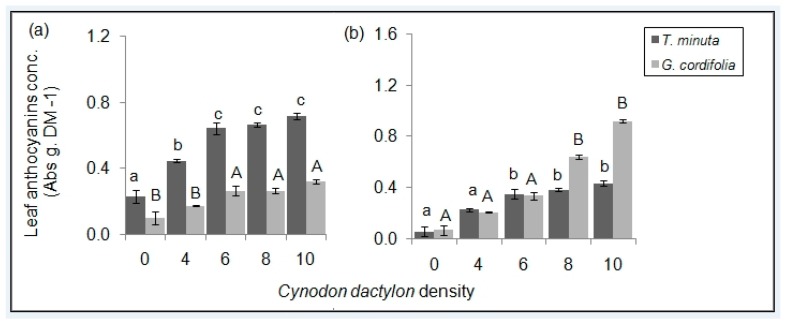
Mean (±SE) total leaf anthocyanins of *T. minuta* and *G. cordifolia* planted with various *C. dactylon* densities (**a**) in the screen house and (**b**) field plot experiments. *C. dactylon* densities ranged from 0 to 10, while the number of weeds per pot/plot was two.

Leaf anthocyanin concentrations differed significantly across the five *C. dactylon* treatments in both *T. minuta* and *G. cordifolia* (*p* < 0.05) (Figure 8; Table 1 and Table 2). *T. minuta* and *G. cordifolia* leaf anthocyanin in pots/plots with *C. dactylon* density ≥ 8 per pot/plot were twice as high as those of *T. minuta* and *G. cordifolia* in control pots/plots.

## 3. Discussion

### 3.1. C. dactylon Effects on Invasive Plants Growth Parameters

Our experiments showed that increasing densities of *C. dactylon* affected *T. minuta* and *G. cordifolia* growth negatively both in field trials (plot experiments), i.e., under a semi-controlled environment, as well as in the screen house trials (pot experiments). Increasing densities of *C. dactylon* decreased invasive plant height, shoot diameter, shoot biomass, leaf area, root biomass, the number of vegetative branches, leaves and root length. This was strongly visible for *T. minuta*, while in *G. cordifolia*, only shoot diameter, shoot biomass, the number of vegetative branches and leaf area was negatively affected. These growth parameters are crucial to the growth and overall fitness of a plant [pers. Obs.]. Plant height, leaf area, and number of leaves, for instance, are responsible for catching up to a recommended 95% of the incoming solar radiation for photosynthesis [13,14]. In addition, large shoot diameters and high biomass aid in stress tolerance towards, e.g., trampling by animals and wind destruction [15]. The number of panicles per plant determines the number of seeds deposited in the soil, which is crucial for invasion success of most weeds [16]. We observed a significant reduction in the number of *T. minuta* and *G. cordifolia* panicles with increasing density of *C. dactylon*, which suggests that *C. dactylon*, can potentially reduce the invasive seed number and deposition. This highlights the great potential of this technique for managing weeds in affected areas [16,17]. The fact that the negative effects of *C. dactylon* were strongly visible in *T. minuta* versus *G. cordifolia* may be due to *T. minuta*’s shorter and lighter roots compared to those of *G. cordifolia* [pers. obs]. Possibly, *G. cordifolia*’s greater root weight and length render this species less prone to suppression by competition compared to *T. minuta*. This has also been found for other species that had large root biomass. As predicted, the negative competitive effects were more pronounced with increasing densities of *C. dactylon*. *C. dactylon* has been reported to be a very strong competitor to most crops [11], likely due to increased competition for available nutrients and space. Competitiveness of *C. dactylon* has been associated with its stoloniferous nature and its ability to develop deep roots [4] that can easily outcompete other plant species. While monocultures from invasive plants have been reported to not only suppress other native species [18], but also to deteriorate soil health [19] intercrops were shown to be mostly facilitative in nature, especially in maize-legume combinations [20,21]. In our study, inter-planting of *C. dactylon*/*T. minuta* and of *C. dactylon/G. cordifolia* reduced invasive growth performance of the two invasive species, hence, highlighting the potential for utilizing *C. dactylon* as a suppressor for the two invasive plants.

### 3.2. C. dactylon Effects on Invasive Plants Leaf Pigmentations

As *C. dactylon* density increased, leaf chlorophyll content dropped for both *T. minuta* and *G. cordifolia*. This could be due to the weed species’ reduced access to water, nutrients and space. For example, as Nitrogen becomes less available to a particular plant, its chlorophyll production is reduced [22,23], and consecutively, its ability to conduct photosynthesis. Hence, in our study, the increase in *C. dactylon* density might have exerted enough stress to affect the chlorophyll productivity of the two invasives. Leaf chlorophyll content has been linked to plant health status [24] as it is associated with energy production, and hence, important for other metabolic activities [24]. Plants with reduced chlorophyll amount and photosynthetic capacity [23] also possess flowers with accelerated abscission [25], which reduces the chances of dispersal by pollinators. Reduced dispersal of the two invasives will lower the ability of the species to invade as a monoculture, which has been proven to be devastating in an invaded ecosystem [12,18].

We observed an increasing anthocyanin concentration in *T. minuta* and *G. cordifolia* with increasing numbers of *C. dactylon*. Anthocyanins, which are a small group of flavonoid pigments, form red-blue coloration in most plants that indicates elevated stress levels [26]. The increase of anthocyanin levels in plant leaves under increasing *C. dactylon* densities in this study can be linked to the increasing level of competition [27], specifically for nutrients and space. This is in line with [28] who argued that anthocyanin induction and/or accumulation in plant tissue could be associated with nitrogen and/or phosphorus deficiency. It is a known fact that the rate of photosynthesis is directly proportional to plant’s chlorophyll content [29], anthocyanin pigments reduce a plant’s chlorophyll content, thereby negatively affecting photosynthesis. Therefore, we propose that if *C. dactylon* can be sown in areas invaded by *T. minuta* and *G. cordifolia* can potentially be used as an environmentally friendly invasive species management approach. As *C. dactylon* is also a valuable forage grass, its inclusion into rangelands not only suppresses invasives in the long term, but also enhances fodder quality of previously degraded rangelands, act as a cover crop, reduce soil erosion and can improve soil quality by enhancing the organic matter content.

## 4. Materials and Methods

### 4.1. Study Area Location and Climate

Both screen house and plot experiments were conducted within the Nelson Mandela African Institution of Science and Technology (NM-AIST) main campus (Tengeru) located at 3°24’05.44”S, 36°47’43.24”E in Northern Tanzania. The area’s mean annual temperature and precipitation are 19.5 °C and 90 mm, respectively [30]

### 4.2. Study Species

We selected *C. dactylon* as a suppressor species as it has been reported to successfully escape from stresses like invasion and drought by creeping away from invaded areas through stolones and by developing a deep root system [10]. This species can grow on soils with a wide range of pH, survive flooding [12], and can grow twice as large in mixed cultures than in monoculture [31]. The plant has further been reported to be highly competitive over most crops [11], which highlights its importance as a potential fodder grass for management of invasive weeds.

*T. minuta* is an unpalatable exotic invasive plant native to Mexico [32,33]. The species has escaped cultivation in most nations and is considered a noxious weed in various parts of southern Africa [34]. This species has been introduced and became a weed in most rangelands and farmlands of Tanzania [35]. Its seeds and leaves produce some secondary products, which makes it unpalatable to most herbivores [36]. According to [37], *T. minuta* root exudates contain a polyacetylene derivative (thiophene), which can kill root knot nematodes [38]. Therefore, this invasive can possibly affect the growth and development of leguminous pastures that are crucial for improving soil nitrogen in otherwise nutrient-poor tropical and sub-tropical soils.

*G. cordifolia*, on the other hand, is an unpalatable annual plant native to Africa (pers. com). Its leaves and flowers are allergenic and toxic to animals as they contain a chemical sesquiterpene lactone [39,40] that alters the microbial composition of the rumen, thereby, affecting overall metabolic functioning [39]. In Kenya, the plant has already been reported to be an invasive weed in most farmlands [41,42]; whereas, in Tanzania, the species seems to have invaded and dominated more than half of the Ngorongoro crater floor (250 km^2^) in the Ngorongoro Conservation Area, a UNSESCO World Heritage site [43], and most parts of the Serengeti ecosystem (pers. obs).

### 4.3. Experimental Design

We selected our study species during a field survey in the year 2015/16 to assess the effect of invasion status of *T. minuta* and *G. cordifolia* on the diversity and abundance of native plant species in the Ngorongoro Conservation Area [44]. The results of this survey indicated that *C. dactylon* was the most abundant and coexisting native grass species that grew together with the two invasive species, *T. minuta* and *G. cordifolia*. Despite its ability to compete with *T. minuta* and *G. cordifolia C. dactylon* has been ranked as one of the best forage species utilized by herbivores, due to its high pasture agronomic values [45]. This prompted us to select *C. dactylon* as competitor species and assessed its effects on growth parameters and leaf pigments of *T. minuta* and *G. cordifolia* in both screen house and field plot experiments. We followed a common garden design by varying *C. dactylon* densities during the rainy season (February- May 2016). The minimum and maximum mean monthly temperatures during experiment time were 15.3 °C and 25.2 °C, respectively. We hypothesized that *C. dactylon* could suppress the two weeds, and therefore, reduce their growth and development through suppression of the studied parameters.

**Figure 9:**
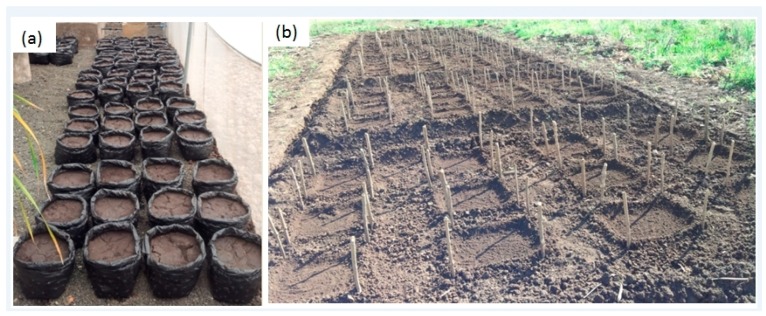
Pots (**a**) and plots (**b**) used in both screen house and field experiments, respectively, at the NM-AIST.

Both *T. minuta* and *G. cordifolia* seeds were collected from the Ngorongoro Crater within the Ngorongoro Conservation Area (NCA). The collected *T. minuta* and *G. cordifolia* seeds were sown separately both in pots (screen house) and in plots (field), and in combination with *C. dactylon* following a simple additive design [46] in early 2016. Clay-loam soil was used in both experiments, which is similar to the Ngorongoro Crater’s soil, where the previous field survey [44] had been carried out. In the screen house, both weed species *T. minuta* and *G. cordifolia* were grown in combination with *C. dactylon* in pots of 0.15 m height × 0.56 m diameter and plots of 0.50 m × 0.50 m in the field (Figure 9).

**Figure 10:**
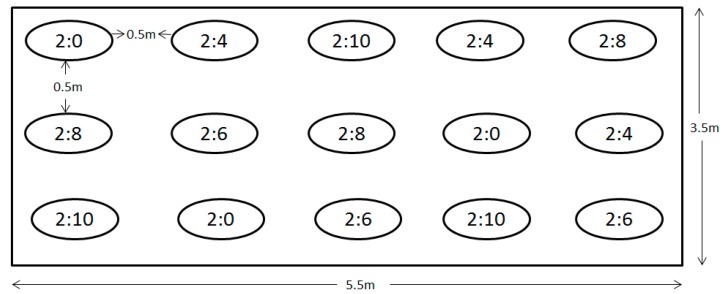
Experimental pot/ plots layout. Different ratios represent proportions of sown weeds (2) to the suppressor *C. dactylon* (0, 4, 6, 8 and 10).

Based on the used pot and plot size, density proportions of sown weeds (*T. minuta* and or *G. cordifolia*) versus *C. dactylon* were as follows: 2:0 (weed: *C. dactylon*), 2:4, 2:6, 2:8 and 2:10, whereby 2:0 was used as control and each treatment was replicated three times (Figure 10). A total of 30 pots (five densities, three replications and two species) and 30 plots (five densities, three replications and two species) were used in this study. The interaction between *T. minuta*, *G. cordifolia* and *C. dactylon* under uniform conditions (space, moisture and nutrients) was studied using a completely randomized design. Seeds of *T. minuta*, *G. cordifolia* and *C. dactylon* were sown at a spacing of ≥ 2 cm apart. During the first two weeks, 100% of planted seeds germinated. During this period, pots/plots were irrigated with water ad-libitum to ensure seedling establishment. After successful establishment, plants in all pots/plots were irrigated with 0.5 l of water daily, and watering ceased at anthesis, which marked the end of the vegetative growth phase (meristematic and elongation growth) of both weed species (at nine weeks).

### 4.4. Growth Parameters Measured 

The number of vegetative branches, leaves, panicles, and seedling height and shoot diameter was measured as growth parameters indicating the fitness of the two invasive plants. Parameters were measured at the end of the plant’s vegetative growth phase. In this study, the number of weeds per pot/plot was considered as the entire population, 100% of which was sampled (two plants per pot/plot). The number of vegetative branch leaves and panicles were counted for each plant. Plant height was measured using a meter ruler, while shoot diameter was measured using vernier calipers at a height of 5 cm above the ground. The total number of leaves in all pots/plots under the same treatment was considered as a population. Of this, about 30% of leaves were randomly sampled for leaf area determination. Leaf area was determined using java based image processing software Image J [47]. *T. minuta* and *G. cordifolia* root lengths were measured using a meter ruler. Young leaves from the top-most part of the plant were sampled randomly per pot for chlorophyll determination, while mature leaves were randomly sampled for anthocyanin level determination.

During the 9th week, (the end of vegetative growth phase), both *T. minuta* and *G. cordifolia* were harvested (uprooted), washed, placed into paper bags and dried at 80 °C for 48 h [48]. Shoot and root material was separated and weighed to obtain the total above/below ground dry biomass [48].

### 4.5. Measurement of Leaf Pigments 

Leaf chlorophyll content has been linked to plant health status [24], and therefore, represents a crucial parameter to be assessed during plant growth and development. Leaf chlorophyll of *T. minuta* and *G. cordifolia* plants was extracted according to [3], with some modifications—50 mg of fresh leaves of 2.25 cm^2^ were immersed in 4 ml of Dimethyl Sulfoxide (DMSO) and incubated at 65 °C for 12 h. The extract was transferred to glass cuvettes for absorbance determination. The absorbance of blank liquid (DMSO) and samples were determined under 2000 UV/VIS spectrophotometer (UNICO^®^) at 663 nm and 645 nm [3], and the total leaf chlorophyll (total Chl) calculated according to [49] using the following equation:

*Total Chl* = 0.0202*A*_663_ + 0.00802*A*_645_

where *A*_663_ and *A*_645_ are absorbance readings at 663 nm and 645 nm respectively.

A bioassay of anthocyanin levels in leaves of *T. minuta* and *G. cordifolia* was performed as described by [50]. Leaves of *T. minuta* and *G. cordifolia* were oven-dried at 60 °C for 48 h, weighed, and ground into a fine powder. Then, 0.10 g of leaf powder was weighed and mixed with 10 ml of acidified methanol prepared from a ratio of 79:20:1 MeOH:H_2_O:HCl. The mixture was incubated for 72 h in darkness for auto-extraction and filtered through Whatman paper Number 2. The extract was transferred to glass cuvettes for absorbance determination. The absorbance of acidified methanol as standard and that of samples were determined under a 2000 UV/VIS spectrophotometer (UNICO^®^) at 530 nm and 657 nm and expressed as Abs g.DM-1 [50]. Anthocyanin concentration in leaf extracts was calculated using the following equation [50]:

Anthocyanin concentration = A_530_ − 1/3A_657_,

where *A*_530_ and *A*_657_ are absorbance readings at 530 nm and 657nm, respectively

### 4.6. Data Analysis

Shapiro-Wilk test for normality was performed on the number of vegetative branches, leaves, panicles, plant height, shoot diameter, root length, leaf area, leaf total chlorophyll content, leaf anthocyanin concentration, and shoot and root biomass of *T. minuta* and *G. cordifolia*. For all data that passed normality test, one-way analysis of variance (ANOVA) was carried out whilst for non-normally distributed data, a Kruskal–Wallis test was performed. For both invasive weed species, one-way ANOVA was performed on the number of vegetative branches, number of panicles, leaf area, shoot diameter, leaf total chlorophyll and leaf anthocyanins concentration versus varying density of *C. dactylon*. Kruskal-Wallis test was carried out on the number of leaves, plant height, root biomass, shoot biomass and root length per plant. Pearson’s Product Moment and Spearman correlations were also performed on normally and non-normally distributed data, respectively. The resulting means were separated by the Fisher’s Least Significant Difference (LSD). The statistical software used was STATISTICA version 8, and the level of significance was set at *p* < 0.05.

## 5. Conclusions 

We found a shorter plant height, smaller shoot diameter, smaller leaf area and lower shoot biomass of *T. minuta* and *G. cordifolia* under higher *C. dactylon* densities, which significantly reduced the vigor of both invasive species. Moreover, reduced leaf total chlorophyll and increased anthocyanin levels in leaves likely affected the photosynthetic ability of both invasive species. Hence, in this study, we highlight the ability of *C. dactylon* to suppress *T. minuta* and *G. cordifolia* under controlled conditions. Furthermore, our results showed that the critical density of *C. dactylon* to suppress the two invasive species is ≥ 8 plants/m^2^. Our findings are important in developing alternative eco-friendly ways to suppress weeds, particularly in rangelands and protected areas where conventional methods, such as chemical applications must be limited.

## Figures and Tables

**Table 1:** Effects of increasing densities of *C. dactylon* on *T. minuta* and *G. cordifolia* growth parameters and leaf pigmentation in the screen house experiment.

Parameters	*T. minuta*				*G. cordifolia*			
	MS	*F*	*H*	*P*	MS	*F*	*H*	*P*
Veg. branches per/plant	-	-	10.0	0.03	46	4.4	-	0.02
Plant height (cm)	1732	32.2	-	<0.01	452	1.9	-	0.18
Leaf area (mm^2^)	-	-	12.2	0.01	1213	6.6	-	0.01
No. of leaves/plant	162	23.9	-	<0.01	12	1.3	-	0.33
Shoot diameter (mm)	19	21.6	-	0.01	8	8.2	-	<0.01
Shoot biomass (g)	-	-	11.7	0.02	-	-	11.1	0.02
Root biomass (g)	-	-	12.2	0.01	-	-	1.3	0.86
Root length (cm)	142	5.2	-	0.02	44	1.9	-	0.17
No. of panicles/plant	526	68.7	-	3.1 × 10^−7^	201	37.3	-	5.6 × 10^−6^
Chlorophyll (unit?)	0.03	116.6	-	<0.01	-	-	13.2	0.01
Anthocyanins (Abs g.DM^−1^)	-	-	11.7	0.02	0.01	2.5	-	0.11

*MS = Mean square, F = F-value, H = Kruskal-Wallis H-test, P = *p*-value.

**Table 2:** Effects of increasing densities of *C. dactylon* on *T. minuta* and *G. cordifolia* growth parameters and leaf pigmentation in the field plot experiment.

Parameters	*T. minuta*				*G. cordifolia*			
	MS	*F*	*H*	*P*	MS	*F*	*H*	*P*
Veg. branches per/plant	379	65.1	-	<0.01	184	14.3	-	<0.01
Plant height (cm)	1414	13.3	-	0.01	480	1.7	-	0.22
Leaf area (mm^2^)	43,487,857	31.4	-	<0.01	-	-	12.3	0.02
No. of leaves/plant	206	17.4	-	<0.01	67	7.9	-	0.01
Shoot diameter (mm)	35	38.7	-	<0.01	9.7	32.8	-	<0.01
Shoot biomass (g)	-	-	13.5	0.01	-	-	10.5	0.03
Root biomass (g)	-	-	13.2	0.01	-	-	7.0	0.13
Shoot biomass (g)	-	-	13.5	0.01	-	-	10.5	0.03
Root length (cm)	250	13.8	-	<0.01	54.8	2.8	-	0.08
No. of panicles/plant	439	41.6	-	3.3 × 10^−6^	-	-	12.8	0.01
Chlorophyll (..)	-	-	13.0	0.01	-	-	13.5	0.01
Anthocyanins ( Abs g.DM^−1^)	0.0676	28.7	-	<0.01	0.024	12.5	-	0.01

*MS = Mean square, F = F-value, H = Kruskal-Wallis H-test, P = *p*-value.
